# Autophagic flux is required for the synthesis of triacylglycerols and ribosomal protein turnover in *Chlamydomonas*

**DOI:** 10.1093/jxb/erx372

**Published:** 2017-10-19

**Authors:** Inmaculada Couso, María Esther Pérez-Pérez, Enrique Martínez-Force, Hee-Sik Kim, Yonghua He, James G Umen, José L Crespo

**Affiliations:** 1 Instituto de Bioquímica Vegetal y Fotosíntesis, Consejo Superior de Investigaciones Científicas (CSIC)-Universidad de Sevilla, Seville, Spain; 2 Instituto de la Grasa (CSIC), Edificio 46, Campus Universitario Pablo de Olavide, Seville, Spain; 3 Korea Research Institute of Bioscience and Biotechnology (KRIBB), Daejeon, Korea; 4 Donald Danforth Plant Science Center, St. Louis, MO, USA; 5 Swedish University of Agricultural Sciences, Sweden

**Keywords:** Autophagy, *Chlamydomonas*, concanamycin A, lipid body, nitrogen, phosphate, ribosomal protein, triacylglycerol

## Abstract

Autophagy is an intracellular catabolic process that allows cells to recycle unneeded or damaged material to maintain cellular homeostasis. This highly dynamic process is characterized by the formation of double-membrane vesicles called autophagosomes, which engulf and deliver the cargo to the vacuole. Flow of material through the autophagy pathway and its degradation in the vacuole is known as autophagic flux, and reflects the autophagic degradation activity. A number of assays have been developed to determine autophagic flux in yeasts, mammals, and plants, but it has not been examined yet in algae. Here we analyzed autophagic flux in the model green alga *Chlamydomonas reinhardtii*. By monitoring specific autophagy markers such as ATG8 lipidation and using immunofluorescence and electron microscopy techniques, we show that concanamycin A, a vacuolar ATPase inhibitor, blocks autophagic flux in *Chlamydomonas*. Our results revealed that vacuolar lytic function is needed for the synthesis of triacylglycerols and the formation of lipid bodies in nitrogen- or phosphate-starved cells. Moreover, we found that concanamycin A treatment prevented the degradation of ribosomal proteins RPS6 and RPL37 under nitrogen or phosphate deprivation. These results indicate that autophagy might play an important role in the regulation of lipid metabolism and the recycling of ribosomal proteins under nutrient limitation in *Chlamydomonas*.

## Teaser

Inhibition of autophagic flux prevented the synthesis of triacylglycerols, formation of lipid bodies, and degradation of ribosomal proteins RPS6 and RPL37 in nitrogen- or phosphate-starved *Chlamydomonas* cells.

## Introduction

Eukaryotic cells have developed specialized mechanisms to respond properly and adapt to perturbations in the extracellular environment. The process of autophagy is a well-characterized case of such stress-responsive mechanisms. Autophagy is a catabolic process by which damaged or unnecessary cytoplasmic material is engulfed in bulk by double-membrane vesicles called autophagosomes and delivered to the vacuole or lysosome for degradation and recycling (He and Klionsky, 2009; Mizushima *et al.*, 2011; Li and Vierstra, 2012; Liu and Bassham, 2012). Autophagy occurs constitutively at a low basal level, but various stress conditions including nutrient starvation, oxidative damage, and organelle deterioration up-regulate this degradative process in order to maintain cellular homeostasis (Li and Vierstra, 2012; Liu and Bassham, 2012). In plants, it has been shown that autophagy plays a critical role in programmed cell death during normal development and during the hypersensitive response triggered by pathogen infection (Hofius *et al.*, 2009; Minina *et al.*, 2013*a*). Initially considered as a non-selective degradation process, autophagy was later demonstrated to clear selectively certain organelles and protein aggregates in yeasts, mammals, and plants (Kraft *et al.*, 2009; Floyd *et al.*, 2012; Li and Vierstra, 2012; Michaeli *et al.*, 2016; Kellner *et al.*, 2017). For instance, removal of damaged mitochondria via mitophagy, inactive proteasomes via proteaphagy, or photo-damaged chloroplasts via chlorophagy has been reported among other selective forms of autophagy (Kanki *et al.*, 2009; Okamoto *et al.*, 2009; Marshall *et al.*, 2015; Izumi *et al.*, 2017).

Autophagy was first described in mammalian cells using electron microscopy (De Duve and Wattiaux, 1966), but pioneering work performed in the model yeast *Saccharomyces cerevisiae* settled the molecular basis of this catabolic process. Autophagy is mediated by a set of proteins coded by autophagy-related (*ATG*) genes, which were identified through genetic screens for autophagy-defective mutants in yeasts (Tsukada and Ohsumi, 1993). The high sequence conservation in the eukaryotic lineage of many of these genes allowed the identification of *ATG* orthologs in the genome of other eukaryotes including plants and algae (Thompson and Vierstra, 2005; Bassham *et al.*, 2006; Diaz-Troya *et al*., 2008). A group of ATG proteins constitute the core autophagy machinery and are required for the formation of the autophagosome and its fusion to the vacuole (Xie and Klionsky, 2007; Mizushima *et al.*, 2011; Feng *et al.*, 2014). This group of proteins includes the ATG8 and ATG12 ubiquitin-like proteins required for phagophore expansion. The ATG8 protein has been widely used to monitor autophagy in many systems (Klionsky *et al.*, 2016) because, unlike other ATG proteins, ATG8 firmly binds to the autophagosome membrane through a covalent bond to phosphatidylethanolamine (PE) in a process known as ATG8 conjugation or lipidation (Mizushima *et al.*, 2011). Detection of lipidated ATG8 (ATG8-PE) has proven to be an effective method to monitor autophagy since this modified form of the protein accumulates under conditions that trigger this process. However, an increase in the abundance of ATG8 and/or ATG8-PE does not necessarily reflect increased autophagic flux since a blockage in the process of autophagosome formation may also result in the accumulation of lipidated ATG8 (Klionsky *et al.*, 2016). Thus, the use of autophagy markers such as ATG8-PE needs to be complemented by assays to estimate overall autophagic flux through the entire system. A simple method to determine autophagic flux that has been widely used in yeast and mammalian cells is based on the analysis of ATG8-PE turnover in the absence of vacuolar degradation (Klionsky *et al.*, 2016). Inhibition of vacuolar degradation can be achieved through the use of compounds such as concanamycin A that neutralize the vacuolar pH (Drose *et al*., 1993; Matsuoka *et al.*, 1997) or with agents that inhibit vacuolar proteases (Klionsky *et al.*, 2016). The transit of ATG8-PE through the autophagic pathway has been estimated by analyzing the amount of ATG8-PE in the presence or absence of such inhibitors since an increase of ATG8-PE in the presence of the inhibitor indicates that flux (to the stage of cargo reaching the vacuole) is occurring (Klionsky *et al.*, 2016). Other approaches to investigate autophagic flux have been reported in yeasts and mammals, including flow cytometry or fluorescence microscopy in combination with novel autophagy probes (Klionsky *et al.*, 2016). In plants, autophagic flux has been monitored through the use of fluorescent protein fusions to ATG8 such as green fluorescent protein (GFP)–ATG8 to label autophagosome processing specifically. However, the detection of ATG8-decorated autophagosomes in plants requires pre-treatment with concanamycin A to inhibit vacuolar degradation due to the high turnover rate of autophagosomes in these organisms (Yoshimoto *et al.*, 2004; Thompson *et al.*, 2005; Xiong *et al.*, 2007). The GFP–ATG8 processing assay has also been successfully used to determine autophagic flux in plants (Chung *et al.*, 2009; Suttangkakul *et al.*, 2011).

Most of the ATG proteins that make up the autophagy core machinery are conserved in land plants (Thompson and Vierstra, 2005; Bassham *et al.*, 2006; Avin-Wittenberg *et al.*, 2012) and in evolutionarily distant algae, including freshwater species, such as the model green alga *Chlamydomonas reinhardtii*, and marine species (Diaz-Troya *et al*., 2008; Perez-Perez and Crespo, 2014; Shemi *et al.*, 2015). In contrast to plants, most *ATG* genes including *ATG8* are in single copy in the *Chlamydomonas* genome, which simplifies the study of autophagy in this unicellular alga. Our current knowledge about autophagy in algae is limited compared with other systems, in part due to the lack of specific autophagy markers in these organisms. The generation of an antibody against the ATG8 protein from *Chlamydomonas* has been a fundamental tool to investigate the process of autophagy in this model system (Perez-Perez *et al*., 2010). By monitoring the abundance, lipidation state, and cellular distribution of the ATG8 protein in *Chlamydomonas*, it has been shown that autophagy is elicited under various stress conditions such as nitrogen or carbon deprivation (Perez-Perez *et al*., 2010; Goodson *et al.*, 2011; Davey *et al.*, 2014; Goodenough *et al.*, 2014). Progression into stationary growth phase also activates autophagy in a reversible manner since the process is down-regulated when *Chlamydomonas* cells return to the exponential growth phase (Perez-Perez *et al*., 2010). Mounting evidence revealed that reactive oxygen species (ROS) are potent inducers of autophagy in algae (Perez-Perez *et al*., 2012*b*). Oxidative stress, photo-oxidative damage generated by carotenoid deficiency, high light stress, or the accumulation of unfolded proteins in the endoplasmic reticulum resulted in activation of autophagy in *Chlamydomonas* (Perez-Perez *et al*., 2010, 2012*a*; Perez-Martin *et al*., 2014). Moreover, loss of chloroplast integrity due to depletion of the chloroplastic ClpP protease has been shown to activate autophagy in this model alga (Ramundo *et al.*, 2014). Recent studies have also linked this catabolic process with the degradation of lipid droplets in the green alga *Auxenochlorella protothecoides* (Zhao *et al.*, 2014) or with the propagation of DNA viruses in the marine alga *Emiliania huxleyi* (Schatz *et al.*, 2014).

Despite the increasing data indicating that the autophagic machinery is up-regulated in response to different stress conditions in algae, flux through the entire pathway has not been shown in these organisms. In this study, we show that concanamycin A blocks autophagic flux in *Chlamydomonas* cells. Our results indicated that inhibition of autophagic flux prevents the degradation of ribosomal proteins in nitrogen- or phosphate-starved cells, strongly suggesting that these proteins are cleared via autophagy upon nutrient limitation. Furthermore, we found that vacuolar lytic function is needed for the synthesis of triacylglycerol (TAG) and lipid bodies in *Chlamydomonas* cells subjected to nitrogen or phosphate starvation.

## Materials and methods

### Strains, media, and growth conditions

*Chlamydomonas reinhardtii* WT 4A+ (CC-4051) was obtained from the Chlamydomonas Resource Center (http://www.chlamycollection.org). *Chlamydomonas* strain OL-Rps6 expressing OLLAS-tagged RPS6 was generated in this study as described below. *Chlamydomonas* cells were grown under continuous illumination at 25 °C in Tris-acetate phosphate (TAP) medium as described (Harris, 1989). When required, cells in exponential growth phase (10^6^ cells ml^–1^) were treated with concanamycin A (Santa Cruz Biotechnology, sc-202111A), wortmannin (Santa Cruz Biotechnology, sc-3505), or 3-methyladenine (3-MA; Sigma, M9281), or subjected to nitrogen or phosphate limitation.

### Epitope tagging of *Chlamydomonas reinhardtii* RPS6 and generation of the OLLAS-RPS6 strain

*Chlamydomonas* gDNA was isolated according to Crespo *et al.* (2005) and used as a template for PCR amplification of three fragments containing the complete *RPS6* gene (the primers used are given in Supplementary Table S1 at *JXB* online). The PCR products were gel purified and cloned into pUC19 using an In-Fusion HD Kit (Clontech, USA) following the manufacturer’s instructions. The OLLAS tag (Park *et al.*, 2008) was inserted into the RPS6 N-terminus using primers rpS6_OLLn_F and rpS6_OLLn_R (Supplementary Table S1). OLLRps6-1R and OLLRps6-2F share an overlapping sequence that encodes the OLLAS tag (Supplementary Table S1, underlined). pKSaphVIII (Sizova *et al.*, 2001), which confers resistance to paromomycin, was used together with the previous construct to co-transform wild-type *Chlamydomonas* cells. Positive clones expressing OLLAS-tagged RPS6 were selected by western blot analysis of paromomycin-resistant clones with an anti-OLLAS antibody. A single band with the expected molecular mass (38 kDa) was detected by western blot in total extracts from *Chlamydomonas* cells expressing OLLAS-tagged RPS6 with the anti-OLLAS antibody (Supplementary Fig. S1). A cross-reacting band was also observed in wild-type cells with the same antibody (Supplementary Fig. S1).

### Protein preparation and immunoblot analysis

*Chlamydomonas* cells from liquid cultures were collected by centrifugation (4000 *g* for 5 min), washed in 50 mM Tris–HCl (pH 7.5) buffer, and resuspended in a minimal volume of the same solution. Cells were lysed by two cycles of slow freezing to –80 °C followed by thawing at room temperature. The soluble cell extract was separated from the insoluble fraction by centrifugation (15 000 *g* for 20 min) in a microcentrifuge at 4 °C. For immunoblot analyses, total protein extracts (20 µg) were subjected to 12% or 15% SDS–PAGE and then transferred to nitrocellulose membranes (Bio-Rad, 162-0115). Primary antibodies anti-CrATG8 (Perez-Perez *et al*., 2010), anti-CrFKBP12 (Crespo *et al.*, 2005), anti-OLLAS (Thermo Scientific, MA5-16125), and anti-Rpl37 (Agrisera, AS122115) were diluted 1:3000, 1:5000, 1:1000, and 1:10 000, respectively. Secondary anti-rat (Thermo Scientific, A18866) and anti-rabbit (Sigma, A6154) antibodies were diluted 1:5000 and 1:10 000, respectively, in phosphate-buffered saline (PBS) containing 0.1% (v/v) Tween-20 (Applichem, A4974) and 5% (w/v) milk powder. The Luminata Crescendo Millipore immunoblotting detection system (Millipore, WBLUR0500) was used to detect the proteins. Proteins were quantified with the Coomassie dye binding method (BioRad, 500-0006).

### Electron microscopy

*Chlamydomonas* cells (~2 × 10^6^ cells ml^–1^) treated with concanamycin A for 0, 4, and 8 h were fixed with 2.5% glutaraldehyde in 0.1 M Na-cacodylate buffer at pH 7.4 for 2 h at 25 °C. After fixing, cells were washed five times with the same buffer at 25 °C. Samples were post-fixed in 1% osmium tetraoxide in cacodylate buffer (0.1 M, pH 7.4) for 1 h at 4 °C. After washing, samples were immersed in 2% uranyl acetate, dehydrated through a gradient acetone series (50, 70, 90, and 100%), and embedded in Spurr resin (Spurr, 1969). Semi-thin sections (300 nm thickness) were obtained with a glass knife and stained with 1% toluidine blue for cell localization and reorientation using a conventional optic microscope. Once a suitable block face of the selected area was trimmed, several ultrathin sections (70 nm) were obtained using an ultramicrotome (Leica UC7) equipped with a diamond knife (Diatome) and collected on 200 mesh copper grids. Sections were examined in a Zeiss Libra 120 transmission electron microscope and digitized (2048 × 2048 × 16 bits) using an on-axis mounted TRS camera.

### Immunofluorescence microscopy

*Chlamydomonas* untreated (control 0 h) or concanamycin A-treated (2, 4, or 8 h) cells were fixed and stained for immunofluorescence microscopy as previously described (Crespo *et al.*, 2005). A purified anti-CrATG8 antibody (Perez-Perez *et al*., 2010) was used at 1:500 final dilution. For signal detection, fluorescein isothiocyanate (FITC)-labeled goat anti-rabbit antibody (Sigma, F4890; 1:500 dilution) was used. Preparations were photographed on a DM6000B microscope (Leica) with an ORCA-ER camera (Hamamatsu) and processed with the Leica Application Suite Advanced Fluorescence software package (Leica). For comparative analysis, the same acquisition time was fixed for the FITC signals.

### Nile red staining

Cells were fixed on ice for 20 min with 2% paraformaldehyde (Sigma-Aldrich, 158127) and then washed twice with PBS buffer. Lipid body staining was performed as described (Wang *et al.*, 2009). Microscopy was performed with a Leica DM6000B (Leica) using a ×100 oil immersion objective with DIC optics or wide field fluorescence equipped with a Leica L5 filter cube (excitation bandpass 480/40 nm; dichroic 505 nm; emission bandpass 527/30 nm) and an ORCA-ER camera (Hamamatsu).

### Flow cytometry

Cells were fixed and stained with Nile red as described above. Samples were subjected to analysis using FL1 (530/30) to score the content of neutral lipids in the cells with a flow cytometer (BD FACSCalibur Cytometry System). Data were processed with CellQuest ProV5.2.1 software (BD; CA, USA). Each measurement was normalized using the corresponding unstained sample. Mean data and SD were calculated using two biological and technical replicates measuring 10 000 cells each.

### Lipid analysis

Total lipids were extracted as described by Couso *et al*. (2016) with the modification that triheptadecanoic acid was added to the freeze-dried pellet as an internal standard before extraction. Briefly, 4 ml of CHCl_3_:methanol (2:1) were added to ~20 mg of freeze-dried cells and then mixed by vortexing. Samples were heated at 42 °C for 30 min followed by addition of 2.5 ml of 0.1 N HCl:1 M NaCl and additional mixing by vortexing. Samples were centrifuged for 2 min at 500 *g* at room temperature and then the aqueous (upper) phase was discarded. The organic phase was washed twice with ultrapure water and then dried under nitrogen gas. Samples were resuspended in 1 ml of hexane.

TAGs were analyzed as previously described (Fernandez-Moya *et al*., 2000). The analysis of TAGs was carried out by injecting 1 µl aliquots of lipid solutions into the GC system, an Agilent 6890 GC apparatus (Palo Alto, CA, USA), using hydrogen as the carrier gas. The injector and detector temperatures were both 370 °C, the oven temperature was 335 °C, and a head pressure gradient from 70 kPa to 120 kPa was applied. The GC column was a Quadrex Aluminium-Clad 400-65HT (30 m length, 0.25 mm id, 0.1 µm film thickness; Woodbridge, CT, USA), and a linear gas rate of 50 cm s^–1^, a split ratio 1:80, and a flame ionization detector (FID) were used. The TAG species were identified according to Fernandez-Moya *et al*. (2000) and quantified by applying the correction factors reported by Carelli and Cert (1993). Four biological replicates were analyzed for each condition.

## Results

### Effect of concanamycin A on *Chlamydomonas* ATG8

**Fig. 1.**
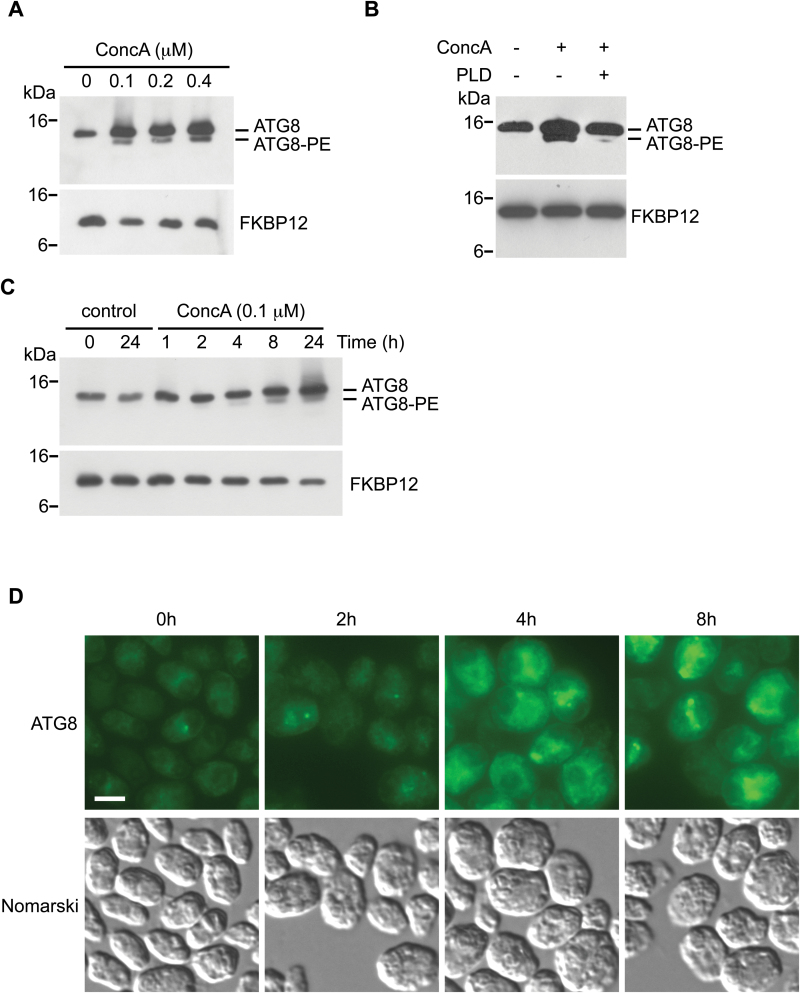
Concanamycin A (ConcA) treatment results in ATG8 accumulation in Chlamydomonas cells. (A) Chlamydomonas cells in exponential growth phase were treated with increasing concentrations (0, 0.10, 0.20, and 0.40 µM) of ConcA for 12 h. (B) Chlamydomonas cells in exponential growth phase were treated with 0.1 µM ConcA for 12 h. Samples of non-treated cells were taken as the same time as a control. A 20 µg aliquot of total extracts from ConcA-treated cells was incubated in the absence (–) or presence (+) of 500 U ml^–1^ phospholipase D (PLD) at 37 °C for 3 h. (C) Chlamydomonas cells in exponential growth phase were treated with 0.1 µM ConcA for various times (1, 2, 4, 8, and 24 h). Samples of non-treated cells were taken at the initial and the latest time (0 and 24 h, respectively) and used as control. For (A), (B), and (C) 20 µg of total extracts were resolved by 15 % SDS–PAGE followed by western blotting with anti-ATG8 and anti-FKBP12 antibodies. The lipidated form of ATG8 (ATG8-PE) is indicated. Molecular mass markers (kDa) are indicated on the left. (D) Immunolocalization of ATG8 in Chlamydomonas cells treated with 0.1 µM ConcA. Chlamydomonas cells growing exponentially were treated with 0.1 μM ConcA for 2, 4, or 8 h. Non-treated cells at 8 h were used as control. Cells were collected and processed for immunofluorescence microscopy analysis with anti-ATG8 antibodies. Scale bar=8 μm.

Concanamycin A, a V-ATPase inhibitor that raises vacuolar pH and impedes hydrolase activity at this cellular compartment (Drose *et al*., 1993; Matsuoka *et al.*, 1997), has been widely used to block autophagic flux in different systems including plants. We investigated the effect of concanamycin A on autophagy in *Chlamydomonas* cells. To this end, we first analyzed whether treatment of log phase cells with different concentrations of concanamycin A may have any effect on *Chlamydomonas* ATG8. Our results indicated that incubation of *Chlamydomonas* cells with 0.1 µM concanamycin A for 12 h led to an increase in ATG8 protein abundance and the detection of a faster migrating band that probably corresponds to ATG8-PE (Fig. 1A). No stronger effects were observed on ATG8 at higher concentrations of concanamycin A (Fig. 1A). To determine the nature of the faster migrating species that accumulated in concanamycin A-treated cells, total extracts were incubated with phospholipase D (PLD), which converts the faster migrating ATG8-PE adduct into the slower migrating free form (Tanida *et al.*, 2004; Fujioka *et al.*, 2008; Chung *et al.*, 2009). In agreement with a previous study showing that PLD solubilizes ATG8 from the membranous fraction in *Chlamydomonas* (Perez-Perez *et al*., 2010), the faster migrating band was sensitive to PLD digestion, indicating that it corresponds to lipidated ATG8 (Fig. 1B). Next, we evaluated the time course effect of 0.1 µM concanamycin A on *Chlamydomonas* ATG8. Modified forms of this protein were detected within 4 h, although the effect was more evident after 8 h of treatment (Fig. 1C). We also investigated the effect of concanamycin A on the cellular distribution of ATG8 by immunofluorescence microscopy using specific antibodies against this protein. As previously shown (Perez-Perez *et al*., 2010, 2012*a*; Perez-Martin *et al*., 2014, 2015), the ATG8 signal was weak in log phase untreated cells and punctate structures could be observed in some cells (Fig. 1D). However, treatment of *Chlamydomonas* cells with 0.1 µM concanamycin A resulted in a progressive increase in ATG8 fluorescence and detection of several spots per cell (Fig. 1D). This effect on ATG8 cellular distribution coincided with the increase in ATG8 abundance and lipidation observed by western blot (Fig. 1C) and may show an accumulation of this protein in the vacuoles due to the inhibition of lytic activity in these cellular compartments. These results indicated that inactivation of vacuolar hydrolases promoted the accumulation and detection of modified ATG8 as well as the localization of this protein at punctate structures in the cell.

**Fig. 2.**
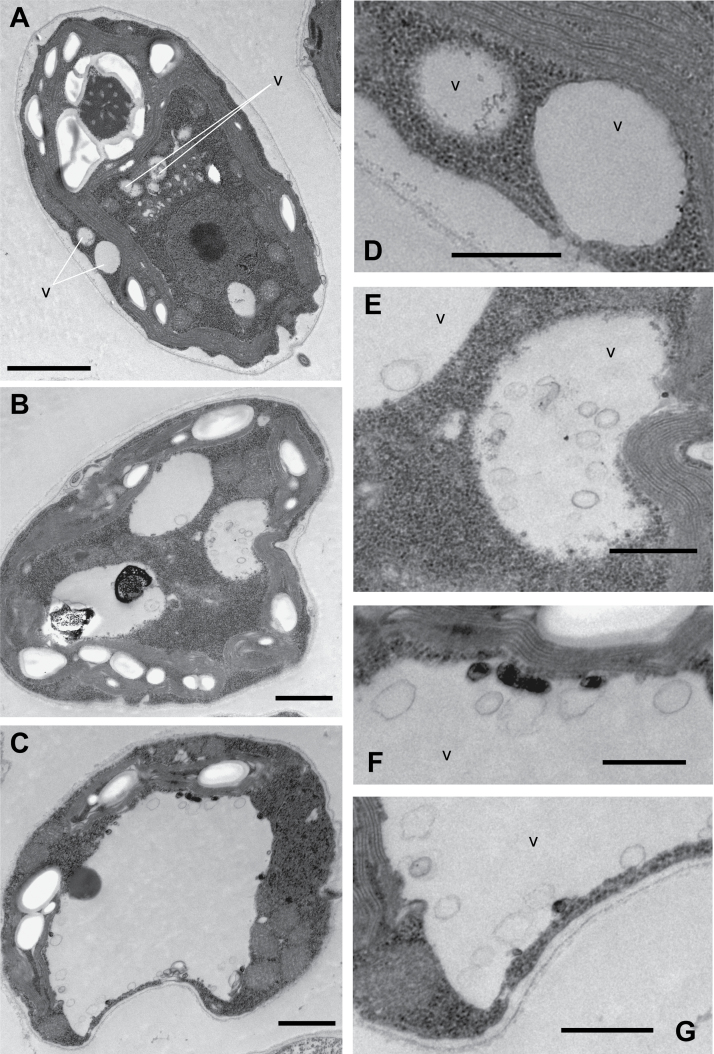
Ultrastructural analysis of Chlamydomonas cells treated with concanamycin A (ConcA). Electron microscopy images from Chlamydomonas cells treated with 0.1 µM ConcA for 0 h (control cells, A), 4 h (B), or 8 h (C). Enlargement of (A), (B), and (C) showing vacuoles of untreated cells (D), ConcA-treated cells for 4 h (E), and 8 h (F and G). v, vacuole. Scale bars=2 µm (A), 1 µm (B, C), 500 nm (D–G).

To characterize further the effect of concanamycin A, we performed an ultrastructural analysis of *Chlamydomonas* cells by electron microscopy. *Chlamydomonas* cells contain a variable number of lytic vacuoles ranging from two to eight and two contractile vacuoles (Sager and Palade, 1957). Lytic vacuoles are frequently found lying between the nucleus and the concave surface of the chloroplast, although they can also be found between the chloroplast and the plasma membrane (Fig. 2A, D) (Sager and Palade, 1957). Treatment of *Chlamydomonas* cells with 0.1 µM concanamycin A for 4 h led to a higher degree of vacuolization and a pronounced increase of vacuole size (Fig. 2B). Moreover, a large, central vacuole could be observed in cells that have been treated for 8 h (Fig. 2C), suggesting that several vacuoles may merge to form a larger one. Remarkably, small vesicles were detected within the vacuoles of concanamycin A-treated cells (Fig. 2E–G). It has been reported that autophagic bodies accumulate in the vacuole of plant cells treated with concanamycin A because vacuolar hydrolases cannot act (Yoshimoto *et al.*, 2004; Thompson *et al.*, 2005; Xiong *et al.*, 2007). Therefore, the small vesicles observed inside the vacuole of *Chlamydomonas* cells treated with this drug probably correspond to autophagic bodies, although a different origin of these vesicles cannot be ruled out. Together, these results indicate that concanamycin A inhibits autophagic flux in *Chlamydomonas*.

### Inhibition of autophagic flux prevents the degradation of ribosomal proteins under nitrogen-limiting conditions

**Fig. 3.**
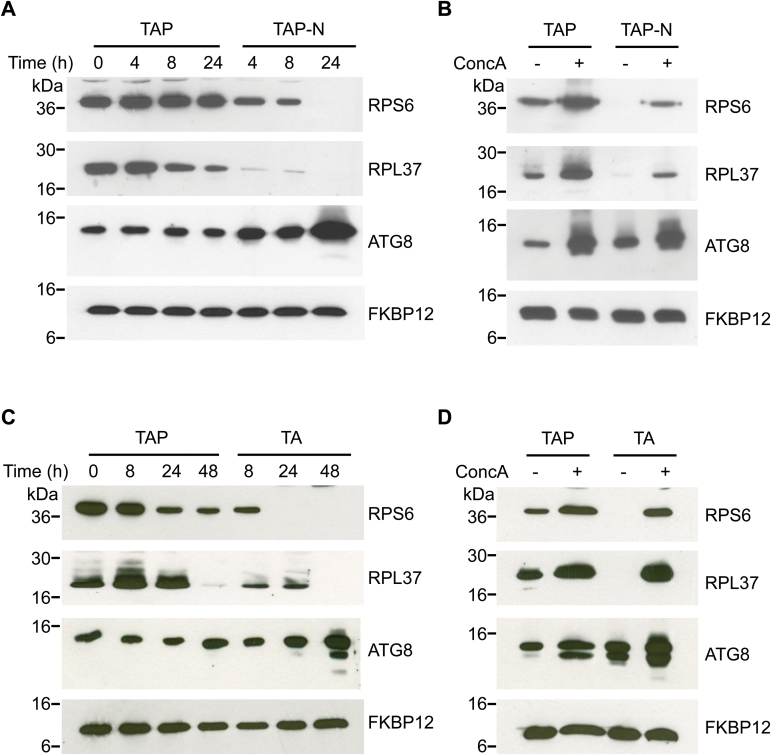
Inhibition of autophagic flux by concanamycin A (ConcA) prevents the degradation of ribosomal proteins under nitrogen starvation or phosphate limitation. (A) Chlamydomonas cells growing exponentially in Tris-acetate phosphate medium (TAP) were washed twice with nitrogen-free medium (TAP-N) and grown under these conditions for 4, 8, and 24 h. Control cells were washed in TAP medium and grown in the presence of nitrogen. (B) Chlamydomonas cells growing in TAP medium were washed with a nitrogen-free medium and grown under these conditions for 16 h in the absence (–) or presence (+) of 0.1 µM ConcA. (C) Chlamydomonas cells growing exponentially in TAP medium were washed with a phosphate-free medium (TA) and grown under these conditions during 8, 24, and 48 h. Control cells were washed and resuspended in TAP medium. (D) Chlamydomonas cells growing in TAP medium were washed with a phosphate-free (TA) medium and grown under these conditions for 48 h. Before collecting samples, cells were treated for 24 h with 0.1 µM ConcA. For (A–D), 20 µg of total extracts were resolved by 12% (RPS6) or 15% (RPL37, ATG8, and FKBP12) SDS–PAGE followed by western blotting with anti-OLLAS, anti-RPL37, anti-ATG8, and anti-FKBP12 antibodies. Molecular mass markers (kDa) are indicated on the left.

Decreased abundance of cytoplasmic and chloroplast ribosomes in nitrogen-limited *Chlamydomonas* cells was documented in the 1970s (Siersma and Chiang, 1971; Martin and Goodenough, 1975), but how ribosomes are degraded remains unknown. We hypothesize that ribosomes are recycled in response to nitrogen starvation via autophagy since this catabolic pathway is active under this nutrient stress condition (Perez-Perez *et al*., 2010). To test this hypothesis, first we analyzed the abundance of two ribosomal proteins, RPS6 and RPL37, in nitrogen-limited cells. To monitor RPS6, we generated a *Chlamydomonas* strain expressing an OLLAS-tagged form of this protein under the control of its own promoter, whereas for RPL37 we used a commercially available antibody against the endogenous protein (see the Materials and methods). Our results revealed that the level of both ribosomal proteins decreased within 4 h of starvation and was almost undetectable after 24 h (Fig. 3A). As previously described (Perez-Perez *et al*., 2010), ATG8 was up-regulated in the absence of nitrogen (Fig. 3A). The removal of ribosomal proteins in nitrogen-starved cells occurs via a reversible process since degradation of RPS6 was quenched within 4–8 h by addition of nitrogen to the medium (Supplementary Fig. S2). Lipidated ATG8 was still abundant at this time, probably due to the high stability of this protein, although it returned to background levels after 24 h of nitrogen repletion (Supplementary Fig. S2). Next we investigated a possible role for autophagy in the down-regulation of RSP6 and RPL37 in nitrogen-stressed cells by blocking autophagic flux with concanamycin A. We observed a substantial increase in the abundance of both ribosomal proteins when *Chlamydomonas* cells grown in nitrogen-rich medium were treated with concanamycin A (Fig. 3B). Moreover, this drug largely prevented the degradation of RPS6 and RPL37 in nitrogen-depleted cells (Fig. 3B). Inhibition of autophagic flux by concanamycin A in these experiments was confirmed by the effect on ATG8 and the detection of lipidated ATG8 (Fig. 3B).

Autophagy can also be blocked at the initiation level by inhibiting phosphoinositide 3-kinase (PI3K) activity, which is required for the formation of the autophagosome. The PI3K inhibitors wortmannin and 3-MA have been widely used to prevent autophagic flux in different organisms including plants (Seglen and Gordon, 1982; Takatsuka *et al.*, 2004). We tested these drugs in *Chlamydomonas* and curiously they failed to prevent the lipidation of ATG8 upon autophagy activation (Supplementary Fig. S3), indicating that wortmannin and 3-MA do not inhibit autophagy in this alga. We also investigated if RPS6 and RPL37 degradation in nitrogen-starved cells could be mediated by the proteasome using MG132, a well-known proteasome inhibitor that has been tested previously in *Chlamydomonas* (Reisdorph and Small, 2004; Dathe *et al.*, 2012). We found that MG132 did not prevent the degradation of RPS6 and RPL37 in nitrogen-stressed cells (Supplementary Fig. S4), strongly suggesting that this degradation is not mediated by the proteasome. Taken together, our results indicated that vacuolar activity may control the level of RPS6 and RPL37 proteins in *Chlamydomonas* and that nitrogen starvation led to the remobilization of these ribosomal proteins via autophagy.

### Vacuolar lytic function is needed for the synthesis of TAGs and lipid bodies under nitrogen-limiting conditions

**Fig. 4.**
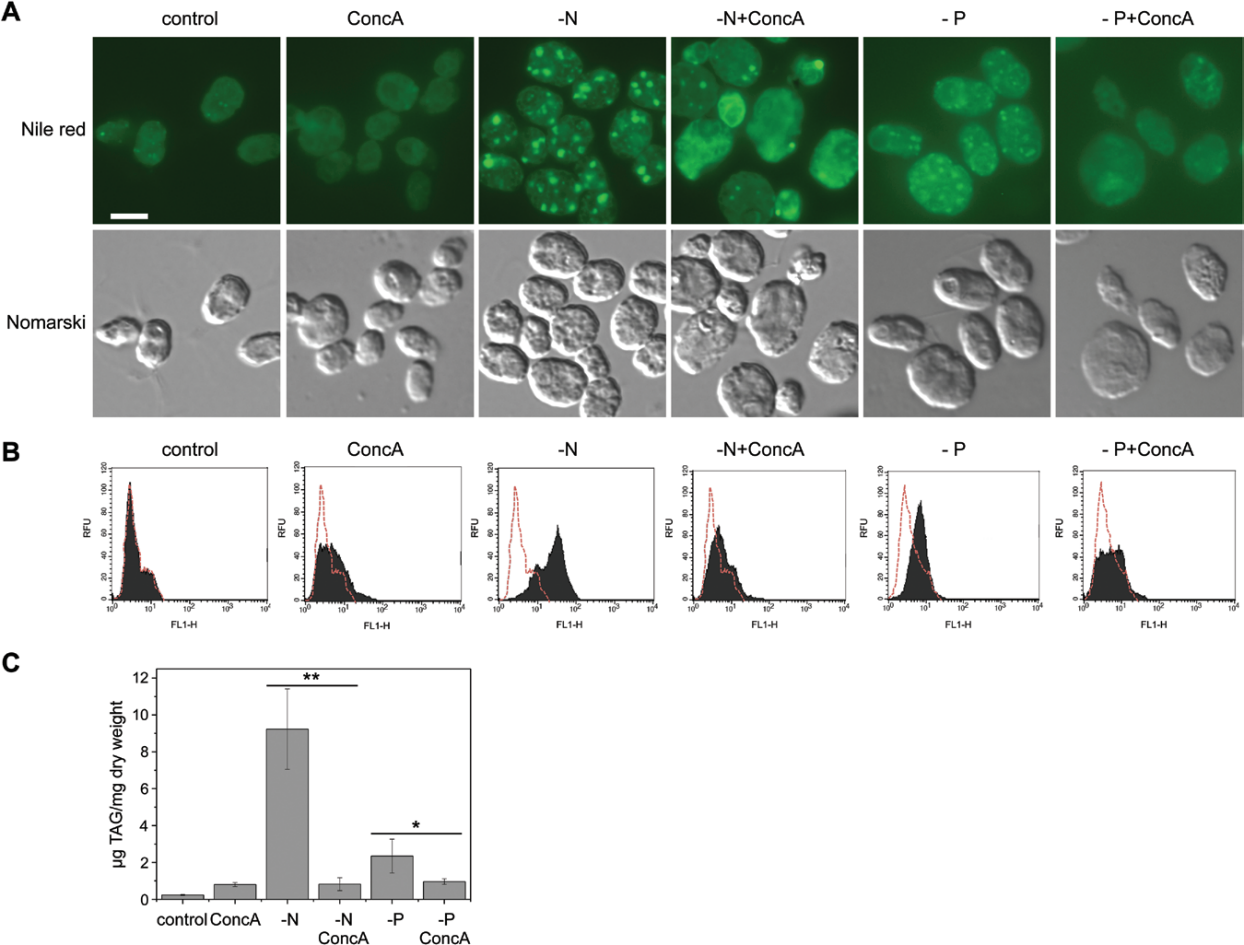
Concanamycin A (ConcA) prevents the formation of lipid bodies and the synthesis of TAGs in nitrogen- or phosphate-limited cells. (A) Chlamydomonas cells growing exponentially in TAP medium were treated as described in Fig. 3B and D for nitrogen or phosphate limitation, respectively, in the absence or presence of 0.1 µM ConcA. Lipid bodies were stained with Nile red and imaged by fluorescence microscopy. Scale bar=8 µm. (B) Lipid bodies from Chlamydomonas cells growing under the same conditions as described in (A) were stained with Nile red and the corresponding fluorescence was analyzed and quantified by flow cytometry (see the Materials and methods). (C) Quantification of triacylglycerols (TAGs) from Chlamydomonas cells subjected to nitrogen or phosphate limitation in the presence of 0.1 µM ConcA. Four biological replicates were analyzed for each condition. **Differences were significant at *P*<0.001 according to the Student’s *t*-test. *P<0.05.

In response to nitrogen limitation, *Chlamydomonas* cells synthesize large amounts of TAGs that are accumulated in specialized structures known as lipid bodies (Wang *et al.*, 2009; Moellering and Benning, 2010; Goodson *et al.*, 2011; Siaut *et al.*, 2011). Given the relevant role that autophagy plays in the cellular response to starvation, we investigated whether this catabolic process is involved in the formation of lipid bodies in nitrogen-depleted cells. To this end, we used concanamycin A to inhibit vacuolar acidification and analyzed the formation of lipid bodies in *Chlamydomonas* cells subjected to nitrogen starvation by staining with Nile red, a reagent that fluoresces upon binding neutral lipids (Elsey *et al.*, 2007). Our results revealed that concanamycin A by itself had no significant effect on lipid body formation, but blocked to a large extent the formation of these structures in nitrogen-starved cells (Fig. 4A). Quantitative analysis of Nile red fluorescence by flow cytometry supported the negative effect of concanamycin A on lipid body formation in nitrogen-starved cells (Fig. 4B). To investigate whether concanamycin A may have a negative effect on TAG synthesis, we determined the level of TAGs in *Chlamydomonas* cells subjected to nitrogen deprivation and autophagy inhibition. We found that the characteristic boost of TAG synthesis in nitrogen-starved cells was fully suppressed by concanamycin A (Fig. 4C), in close agreement with the decreased detection of lipid bodies in these cells. Curiously, concanamycin A treatment under normal growth conditions led to a 2-fold increase in TAG content (Fig. 4C). Together these results indicated that autophagy and vacuolar function might play an important role in the regulation of lipid metabolism as well as in the synthesis of lipid bodies under nitrogen limitation.

### RPS6 and RPL37 are degraded via autophagy under phosphate limitation

In their natural environment, algae and plants often cope with limitations of nutrients other than nitrogen, such as phosphate, the usable form of phosphorus. *Chlamydomonas* has been used as a model system to study the consequences of phosphate depletion in algae (Chang *et al.*, 2005; Moseley *et al.*, 2006; Moseley and Grossman, 2009), but little is known about autophagy or the level of ribosomal proteins under this nutritional stress. Therefore, we decided to investigate whether *Chlamydomonas* cells activate autophagy in response to phosphate limitation. We found that ATG8 is up-regulated when cells were shifted to phosphate-lacking medium, although the effect was moderate and slower compared with nitrogen starvation since the strongest effect was observed after 48 h (Fig. 3C). In addition to ATG8, we also analyzed RPS6 and RPL37 proteins in phosphate-stressed cells. The abundance of these ribosomal proteins decreased within 24 h of phosphate limitation, and both proteins were almost undetectable after 48 h although with different kinetics (Fig. 3C). A decrease in RPS6 and RPL37 proteins was also detected in control cells after 48 h, probably due to the progression of cells into stationary growth (Fig. 3C), which results in autophagy induction (Fig. 3C; Perez-Perez *et al*., 2010). The down-regulation of RPS6 and RPL37 proteins in phosphate-starved cells might be linked to the activation of autophagy in these cells as observed under nitrogen limitation (Fig. 3B). To test this hypothesis, RPS6 and RPL37 abundance was monitored in cells shifted to phosphate-free medium in the presence of concanamycin A. Our results indicated that inhibition of autophagic flux prevented the degradation of both ribosomal proteins under phosphate limitation (Fig. 3D). As shown in nitrogen-starved cells (Fig. 3B), treatment of *Chlamydomonas* cells with concanamycin A in rich (TAP) medium resulted in a pronounced increase in RPS6 and RPL37 abundance (Fig. 3D), suggesting that basal autophagy regulates the level of these ribosomal proteins. The detection of lipidated ATG8 confirmed the inhibition of autophagic flux by concanamycin A in these experiments (Fig. 3D). Taken together, these results show that ribosomal proteins RPS6 and RPL37 can be used to monitor autophagic flux in response to nutrient deprivation in *Chlamydomonas* cells.

### Inhibition of vacuolar lytic function blocks the synthesis of TAGs and lipid bodies in phosphate-limited cells

It has been reported that *Chlamydomonas* cells subjected to prolonged phosphate limitation (7 d) accumulate large amounts of lipid bodies (Bajhaiya *et al.*, 2016). In the present study, we found that *Chlamydomonas* cells respond much faster to phosphate deprivation by activating stress-related processes such as autophagy, which was observed within 48 h (Fig. 3C). Our results revealed that *Chlamydomonas* cells produced detectable levels of lipid bodies by Nile red staining after 48 h in low phosphate medium (Fig. 4A, B). Moreover, we measured the TAG content of phosphate-limited cells and it increased ~8-fold (Fig. 4C). Next, we investigated a possible role for autophagy in the synthesis of lipid bodies in phosphate-starved cells by blocking autophagic flux with concanamycin A. Similar to the effect that we observed in nitrogen-starved cells, inhibition of vacuolar function fully blocked the formation of lipid bodies in phosphate-stressed cells (Fig. 4A, B). In close agreement, we also found that concanamycin A prevented the increased synthesis of TAGs under phosphate limitation (Fig. 4C). These results strongly supported the hypothesis that autophagic flux is required for the synthesis of TAGs and lipid bodies under nutrient stress conditions.

## Discussion

The development of new and specific tools to monitor autophagic flux has been fundamental to investigate the entire process of autophagy. In plants, these techniques include the use of V-ATPase inhibitors such as concanamycin A or bafilomycin A1 in combination with the expression of GFP–ATG8 fluorescence (Yoshimoto *et al.*, 2004; Thompson *et al.*, 2005; Xiong *et al.*, 2007), or the detection of the NBR1 protein level by western blot (Svenning *et al.*, 2011; Zientara-Rytter *et al.*, 2011; Minina *et al.*, 2013*b*). To our knowledge, no assay has been reported to determine autophagic flux in algae. In this study, we show by different approaches that concanamycin A blocks autophagic flux in the model alga *C. reinhardtii*. Our results indicated that a low concentration of concanamycin A (0.1 μM) is sufficient to inhibit autophagy in *Chlamydomonas* cells. Concanamycin A has been widely exploited in the model plant Arabidopsis as an effective inhibitor of autophagic flux, although the concentrations used in this plant are usually ~5–10 times higher compared with the concentration employed in *Chlamydomonas* (Yoshimoto *et al.*, 2004; Thompson *et al.*, 2005; Xiong *et al.*, 2007). Autophagic flux has been efficiently blocked in cultured tobacco BY-2 cells with 0.1 μM concanamycin A (Yano *et al.*, 2015), suggesting that higher concentrations of this drug might be required for the inhibition of autophagic flux in tissues (leaves and roots) or the complete plant. Inhibitors of PI3K such as wortmannin and 3-MA have also been used to block autophagic flux in plants (Takatsuka *et al.*, 2004; Merkulova *et al.*, 2014). However, our results indicate that these drugs do not inhibit autophagic flux in *Chlamydomonas* (Supplementary Fig. S3) probably due to evolutionary divergence of PI3K in this alga, which shares only 33% and 22% identity with its Arabidopsis and yeast homologs, respectively (unpublished).

We found that concanamycin A inhibits growth of *Chlamydomonas* cells at concentrations >0.2 μM, whereas at 0.1 μM the toxicity of this drug is moderate (Supplementary Fig. S5). The effect of concanamycin A on *Chlamydomonas* has never been reported before, but our results are consistent with a previous study showing high sensitivity of the phylogenetically distant green alga *Scherffelia dubia* to 1.5 μM concanamycin A (Becker and Hickisch, 2005). In that study, concanamycin A and brefeldin A, an inhibitor of protein secretion and Golgi function (Nebenfuhr *et al*., 2002), were used to investigate contractile vacuoles in Scherffelia. Treatment of these cells with brefeldin A resulted in a fast and pronounced increase of the size of contractile vacuoles and the formation of a large central vacuole (Becker and Hickisch, 2005). However, these vacuoles did not seem to be acidified (Becker and Hickisch, 2005) and they are probably different in origin and composition from degradative vacuoles targeted by concanamycin A in *Chlamydomonas*. Supporting a functional difference between vacuoles in these two species, contractile vacuoles in *Chlamydomonas* are not sensitive to brefeldin A (Becker and Hickisch, 2005).

As a general rule, autophagic flux can be measured by inferring ATG8-PE turnover by western blot in the presence and absence of vacuolar degradation (Klionsky *et al.*, 2016). According to this method, ATG8-PE abundance should increase in the presence of concanamycin A (Tanida *et al.*, 2005; Klionsky *et al.*, 2016). Our results revealed that ATG8-PE indeed progressively accumulates in *Chlamydomonas* cells treated with concanamycin A (Fig. 1), indicating that this drug inhibits autophagic flux in this alga. Detection of lipidated ATG8 forms following concanamycin treatment has not been a good marker for autophagic flux in plants mainly due to the high complexity of ATG8 proteins in these organisms (nine and five isoforms in Arabidopsis and maize, respectively; Doelling *et al.*, 2002; Chung *et al.*, 2009). Actually, it has been reported that concanamycin A has no measurable effect on ATG8 lipidation in maize (Reyes *et al.*, 2011). As described in yeasts and mammals, one of the most common assays to monitor autophagy in plants is based on the detection of a GFP/RFP–ATG8 fusion, which decorates both the outer and inner membranes of autophagosomes (Klionsky *et al.*, 2016). However, the high turnover rate of autophagosomes in some plants such as Arabidopsis precludes the detection of ATG8-labeled autophagosomes and requires pre-treatment with concanamycin A to prevent the degradation of autophagic bodies inside the vacuole. Ultrastructural analysis of *Chlamydomonas* cells treated with concanamycin A revealed the presence of single-membrane vesicles inside the vacuoles (Fig. 2), probably corresponding to autophagic bodies as reported in yeasts and plants (Takeshige *et al.*, 1992; Yoshimoto *et al.*, 2004). TEM images of *Chlamydomonas* cells also showed a pronounced increase in the size of the vacuoles in response to concanamycin A treatment (Fig. 2). Such an effect has not been described in plants but, interestingly, it has been reported that yeast cells that had no V-ATPase activity due to inhibition by concanamycin A or had deletion of a V-ATPase subunit exhibited a large vacuole phenotype (Peters *et al.*, 2004; Baars *et al.*, 2007). Treatment of yeast cells with rapamycin, a specific inhibitor of the TOR kinase, also results in vacuole expansion and cell size increase (Barbet *et al.*, 1996; Noda and Ohsumi, 1998). A similar effect has been observed in *Chlamydomonas* cells treated with this drug (Crespo *et al.*, 2005), indicating that there is a correlation between both vacuole and cell size in these unicellular organisms. *Chlamydomonas* cells contain a variable number of small vacuoles distributed throughout the cytoplasm that appear similar to lysosomes and have a degradative function (Sager and Palade, 1957; Park *et al.*, 1999; Komine *et al.*, 2000; Goodson *et al.*, 2011). Despite the essential function of the vacuole in maintaining cell homeostasis, this highly dynamic organelle has not been extensively studied in *Chlamydomonas*, and how the number and size of vacuoles are regulated in algae is currently unknown. In yeasts, it has been shown that the V-ATPase is required for both vacuolar fusion and fission processes, and treatment with concanamycin A inhibits fission (Baars *et al.*, 2007). Whether V-ATPase may play a similar role in algae needs to be explored.

Our results indicated that the level of two cytoplasmic ribosomal proteins, RPS6 and RPL37, decreased under nitrogen or phosphate starvation, suggesting that cytoplasmic ribosomes are turned over under nutrient stress (Fig. 3). Initial biochemical and ultrastructural microscopy studies demonstrated that the abundance of ribosomal proteins decreased in *Chlamydomonas* cells subjected to nitrogen limitation (Siersma and Chiang, 1971; Martin and Goodenough, 1975). More recently, a comprehensive proteomic analysis of nitrogen-starved cells revealed that the level of 47 cytoplasmic ribosomal proteins is reduced (Schmollinger *et al.*, 2014). Transcriptomic data from the same study also showed a fast up-regulation of autophagy genes in response to nitrogen starvation, leading to the hypothesis that cytoplasmic ribosomes might be degraded via autophagy in nitrogen-limiting conditions (Schmollinger *et al.*, 2014). In yeasts, ribosomes are targeted to the vacuole in nitrogen-starved cells by ribophagy (Kraft *et al.*, 2008), a specialized form of autophagy for the recycling of ribosomes (Kraft *et al.*, 2009). Recent studies supported that a similar mechanism might operate in plants (for a recent review, see Bassham and MacIntosh (2017). On the one hand, it has been shown that rRNA turnover in Arabidopsis requires the core autophagy genes *ATG5* and *ATG9* under normal growth (Floyd *et al.*, 2015). On the other hand, autophagy-defective mutants from Arabidopsis accumulated more RPS6 and RPL13 proteins than wild-type plants under both high and low nitrogen (Guiboileau *et al.*, 2013). In *Chlamydomonas*, we found that the level of some ribosomal proteins might be regulated via autophagy. Inhibition of autophagic flux by concanamycin A resulted in the accumulation of RPS6 and RPL37 under exponential growth and prevented the degradation of these proteins under nitrogen limitation (Fig. 3B). These results are in agreement with the accumulation and lipidation of ATG8 in nitrogen-starved cells from *Chlamydomonas* (Perez-Perez *et al*., 2010) and strengthen the hypothesis that ribosomal proteins are recycled by autophagy in this model alga (Schmollinger *et al.*, 2014). However, it remains unknown whether the turnover of ribosomal proteins in nitrogen limitation takes place as part of a bulk degradation of cellular components or as a selective ribophagy process under this stress condition.

In this study, we have also shown that phosphate limitation triggers autophagy in *Chlamydomonas* (Fig. 3C). Phosphate, the form of phosphorus available to living organisms, is highly abundant in most ecosystems, but it is usually limiting due to complexation with metals or organic molecules that cannot be assimilated by most organisms. Like nitrogen, phosphate is an essential macronutrient that is needed by different biochemical and cellular processes, and its limitation elicits a suite of responses that have been extensively studied in algae (Moseley and Grossman, 2009). Upon phosphate deprivation, *Chlamydomonas* cells secrete periplasmic phosphatases, cease division, store carbon as lipids and starch, and down-regulate photosynthesis to prevent photodamage (Wykoff *et al*., 1998, 1999; Shimogawara *et al.*, 1999; Moseley *et al.*, 2006; Moseley and Grossman, 2009). In addition to these processes, our results revealed that phosphate limitation also leads to the degradation of some ribosomal proteins probably due to the activation of the autophagy machinery. Indeed, inhibition of autophagic flux by concanamycin A fully blocked the down-regulation of RPS6 and RPL37 in low phosphate, similar to what was observed in nitrogen-starved cells (Fig. 3D). In the absence of nutrients such as nitrogen or phosphate, cells cannot maintain a high rate of protein synthesis and consequently the abundance of ribosomes must be reduced. Moreover, the ribosomes represent an important source of nitrogen, and their degradation and recycling through autophagy ensures growth adaptation to nutrient stress conditions. Our results also indicated that activation of autophagy developed faster in response to nitrogen deprivation compared with phosphate limitation (Fig. 3A, C). This difference is likely to be due to the fact that *Chlamydomonas* cells accumulate large amounts of phosphate inside acidic compartments known as acidocalcisomes. These organelles are storage vesicles characterized by the presence of polyphosphate and pyrophosphate complexed with calcium, and they have been described in a diverse range of organisms including green algae (Moreno and Docampo, 2009; Blaby-Haas and Merchant, 2014).

A well-established feature of nitrogen-starved cells in *Chlamydomonas* is the massive storage of TAGs in specialized structures known as lipid bodies, lipid droplets, or oil bodies. How these specialized compartments are synthesized and regulated in microalgae is still poorly understood, although the growing interest in these organisms as factories of biodiesel precursors (Merchant *et al.*, 2012; Liu and Benning, 2013) has boosted the identification of structural proteins and metabolic enzymes associated with lipid bodies (Wang *et al.*, 2009; Moellering and Benning, 2010; Goodson *et al.*, 2011; Goodenough *et al.*, 2014; Goold *et al.*, 2015; Tsai *et al.*, 2015). Depending on their location in the cell, two types of lipid bodies have been defined in *Chlamydomonas*: cytoplasmic lipid bodies, which are analogous to those found in seed plants, and plastidic lipid bodies, which accumulate in starch-less mutants (Goodson *et al.*, 2011; Siaut *et al.*, 2011; Goold *et al.*, 2015). The number and size of lipid bodies increase when *Chlamydomonas* cells are exposed to different stress conditions, and the size of lipid bodies can increase as the stress persists (Goold *et al.*, 2015). The strongest effect on lipid body formation in *Chlamydomonas* has been found in nitrogen-starved cells. Under this stress, there is a sharp change in metabolism leading to accumulation of TAGs (Wang *et al.*, 2009; Siaut *et al.*, 2011; Boyle *et al.*, 2012; Schmollinger *et al.*, 2014). Autophagy is also up-regulated in *Chlamydomonas* cells subjected to nitrogen limitation (Perez-Perez *et al*., 2010; Davey *et al.*, 2014; Goodenough *et al.*, 2014; this study). This catabolic process is essential to maintain cellular homeostasis by recycling cell components and generating building blocks such as amino acids and fatty acids that will be demanded in the course of starvation (Liu and Bassham, 2012; Avila-Ospina *et al.*, 2014; Havé *et al*., 2017). Our results revealed that inhibition of vacuolar lytic function by concanamycin A treatment in *Chlamydomonas* largely prevented the synthesis of TAGs and decreased the number of lipid bodies under nitrogen or phosphate deprivation (Fig. 4), suggesting that autophagy is needed for the formation of these lipid structures. Microscopy studies have shown that lipid bodies can be very abundant in *Chlamydomonas* and may occupy a significant volume of the cell (Sager and Palade, 1957; Goodson *et al.*, 2011; Goodenough *et al.*, 2014). Building of such structures must be an energy-demanding process that requires the synthesis of specific proteins and lipids. Based on our findings, we hypothesized that the proper recycling of cell material under nutrient limitation may allow the synthesis of TAGs and the formation of lipid bodies. Thus, our study strongly suggests that autophagy may play a key role in the control of lipid homeostasis in algae.

## Supplementary data

Supplementary data are available at *JXB* online.

Table S1. Sequence of the primers used for cloning and tagging RPS6.

Fig. S1. Detection of OLLAS-tagged RPS6 in total extracts from *Chlamydomonas*.

Fig. S2. Degradation of ribosomal proteins under nitrogen limitation is a reversible process.

Fig. S3. The PI3 kinase inhibitors wortmannin and 3-methyladenine have no effect on autophagy in *Chlamydomonas*.

Fig. S4. The proteasome inhibitor MG132 does not prevent the degradation of ribosomal proteins under nitrogen starvation.

Fig. S5. Concanamycin A inhibits growth of *Chlamydomonas*.

## Supplementary Material

Supplementary figures_S1_S5 Table_S1
