# Supplementary material for: Autophagic flux is required for the synthesis of triacylglycerols and ribosomal protein turnover in Chlamydomonas
Source: J Exp Bot. 2017 Oct 19;69(6):1355–67. doi: 10.1093/jxb/erx372 (PMC6018900; doi:10.1093/jxb/erx372)
Supplement: Supplementary figures_S1_S5 Table_S1 [file erx372_suppl_supplementary_figures_s1_s5_table_s1.pdf]

SUPPLEMENTARY FIGURE LEGENDS

Figure S1. Detection of OLLAS-tagged RPS6 in total extracts from Chlamydomonas. Soluble extracts from wild-type Chlamydomonas (WT 4A+) and OLLAS-tagged RPS6 (OL-Rps6) Chlamydomonas strains were resolved by 12 % SDS-PAGE followed by western-blot analysis with the anti-OLLAS antibody. OLLAS-tagged RPS6 is marked on the right. The asterisk indicates an unspecific band that cross-reacts with the anti-OLLAs antibody in wild-type cells. Molecular mass markers (kDa) are indicated on the left.

Figure S2. Degradation of ribosomal proteins under nitrogen limitation is a reversible process. Chlamydomonas cells growing in TAP medium were washed with a nitrogen-free medium and grown under these conditions for 24 h (TAP-N). Then, 10 mM NH_4Cl was added to the medium (+N) and samples were taken at the indicated times. For detection of RPS6, ATG8 and FBBP12 proteins, 20 µg of total extracts were resolved by SDS-PAGE followed by western blotting with the corresponding antibodies. Molecular mass markers (kDa) are indicated on the left.

Figure S3. The PI3P kinase inhibitors wortmannin and 3-methyladenine have no effect on autophagy in Chlamydomonas.
(A) Chlamydomonas cells growing in a N rich medium (TAP) were washed with nitrogen-free medium (TAP-N) and shifted to these conditions for 16 h in the absence (-) or presence (+) of 5 µM wortmannin (Wort). Twenty µg of total extracts were resolved by SDS-PAGE followed by western blotting with anti-OLLAS, anti-ATG8 and anti-FKBP12 antibodies. Molecular mass markers (kDa) are indicated on the left. (B) Wild-type Chlamydomonas cells (WT 4A+) were subjected to 10-fold serial dilutions and spotted onto TAP plates containing the indicated concentrations (0, 2.5, 5, 10 and 20 µM) of wortmannin (Wort). Plates were incubated at 25°C under continuous illumination for 7 days. (C) Chlamydomonas cells growing exponentially in TAP medium were treated with 20 µM norflurazon (NF) and/or 5 µM 3-methyladenine (3-MA) for 48 h. Twenty µg of total extracts were resolved by SDS-PAGE followed by western blotting

with anti-ATG8 and anti-FKBP12 antibodies. Molecular mass markers (kDa) are indicated on the left.

Figure S4. The proteasome inhibitor MG132 does not prevent the degradation of ribosomal proteins under nitrogen starvation. Chlamydomonas cells growing in a N rich medium (TAP) were washed with nitrogen-free medium (TAP-N) and shifted to these conditions for 16 h in the absence (-) or presence (+) of 10 μM MG132. Twenty μg of total extracts were resolved by SDS-PAGE followed by western blotting with anti-OLLAS, anti-RPL37 and anti-FKBP12 antibodies. Molecular mass markers (kDa) are indicated on the left.

Figure S5. Concanamycin A inhibits growth of Chlamydomonas. Wild-type Chlamydomonas cells (WT 4A+) were subjected to 10-fold serial dilutions and spotted onto TAP plates containing the indicated concentrations (0, 0.025, 0.050, 0.075, 0.10 and 0.20 μM) of concanamycin A (ConcA). Plates were incubated at 25°C under continuous illumination for 6 days.

Figure S1

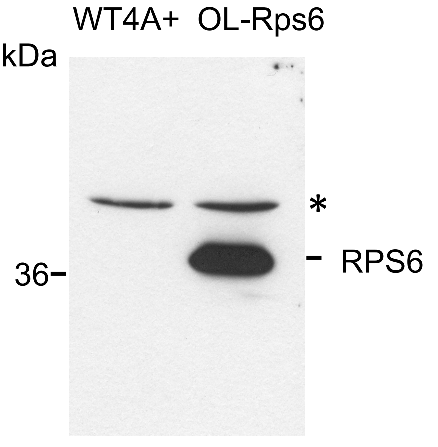

Figure S2

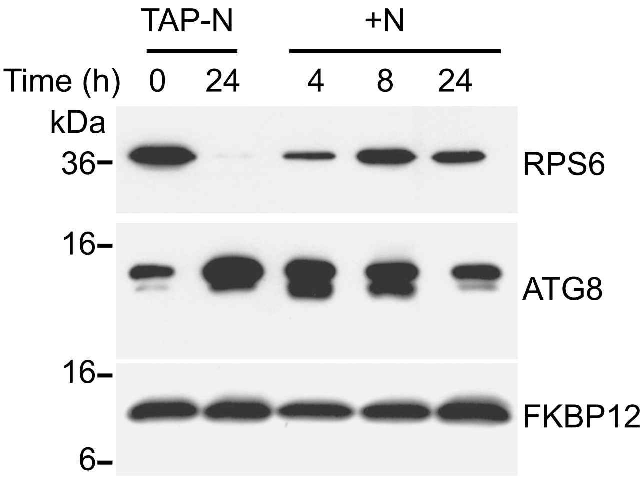

Figure S3

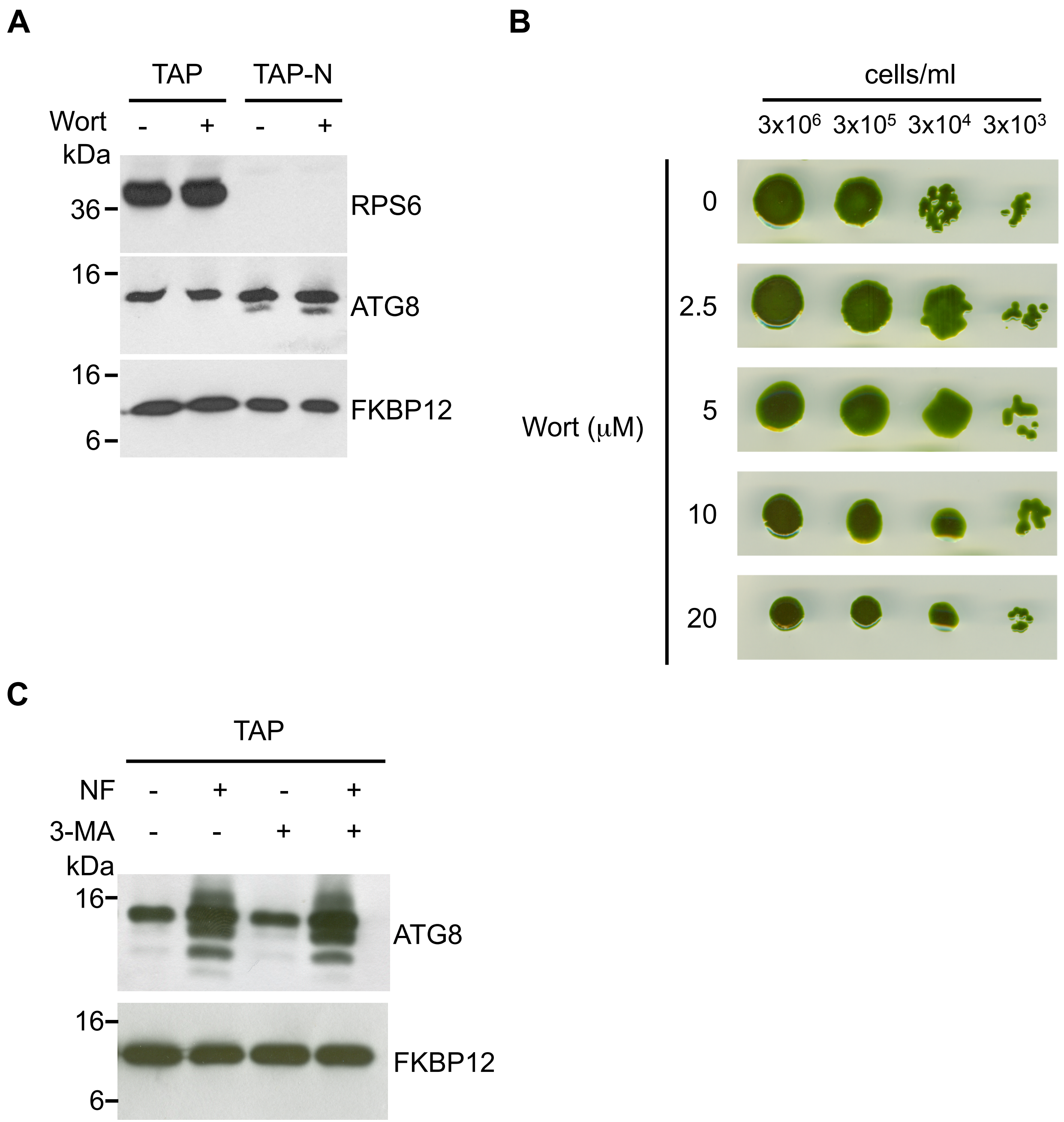
A
TAP
TAP-N
Wort
-
+
-
+
kDa
36
RPS6
16
ATG8
16
FKBP12
6
B
cells/ml
3x10^6 3x10^5 3x10^4 3x10^3
0
2.5
5
10
20
Wort (μM)
C
TAP
NF
-
+
-
+
3-MA
-
-
+
+
kDa
16
ATG8
16
FKBP12
6

Figure S4

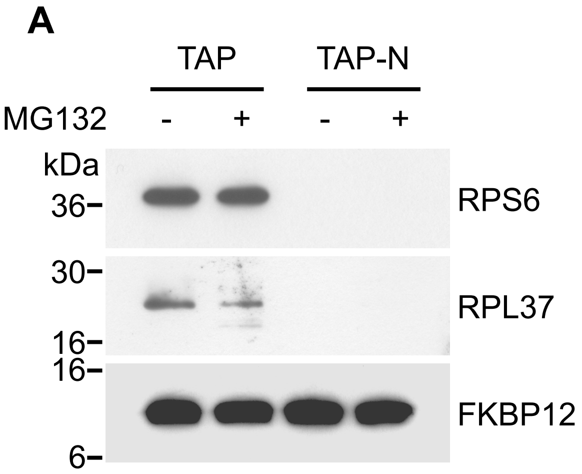

Figure S5

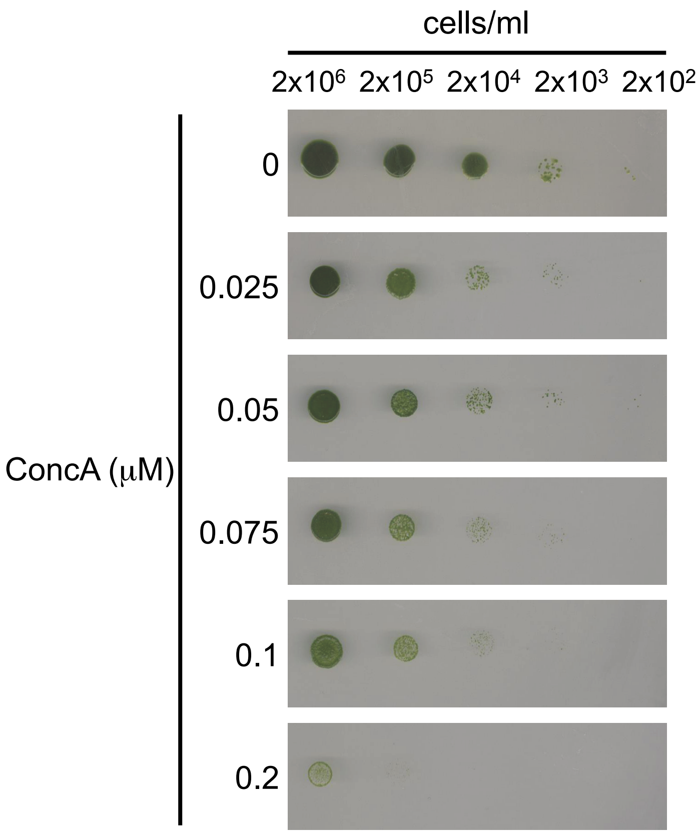
cells/ml
2x10^6 2x10^5 2x10^4 2x10^3 2x10^2
ConcA (μM)
0
0.025
0.05
0.075
0.1
0.2

Table I: Sequence of the primers used for cloning and tagging RPS6. OLLAS tag sequence is underlined.

Name	Sequence (5'-3')	Amplicon size
RPS61F	CGGTACCCGGGGATCCTGGCCCTGCTGATACGCCATCTAACCCCTTGAACC	1.2Kb (2ROLL)
RPS62ROLL	<u>CCTAGGTCCCAATTCATTCGCAAAGCCACTAGT</u>CATTTTTGTTGAAGCTGCTCACCTGCAGCGGCGCA	
RPS63FOLL	<u>GAATTGGGACCTAGGTTGATGGGCAAGCTTTCT</u>AAGCTCAACATTGCCTACCCGGCAACCGGCTG	1.7Kb(4R)
RPS64R	ATGACCAGGTTCAGCACGGCCAGGTCGGGCGACA	
RPS65F	GCTGAACCTGGTCATTGTCAAGAAGGGCGA	2,4Kb(6R)
RPS66R	CGACTCTAGAGGATCCTGCTTAACCTGGGGACTGCGATCCCGCATAGCA	
